# New Insights into a Conceptual Bionic Colonic Bioreactor: A Model, ‘Probiotics in Human Colon’, Showing How Probiotics Alleviate Constipation from a Bioprocess Engineering Perspective

**DOI:** 10.3390/foods14081335

**Published:** 2025-04-12

**Authors:** Ni Wang, Peng Wu, Xiao Dong Chen

**Affiliations:** Life Quality Engineering Interest Group, School of Chemical and Environmental Engineering, College of Chemistry, Chemical Engineering and Material Science, Soochow University, Suzhou 215123, China; p.wu@suda.edu.cn

**Keywords:** probiotics, bionic colonic bioreactor, bioprocess engineering perspective, physical property, constipation

## Abstract

Constipation is a common problem which often causes negative impacts on the patient’s quality of life. Apart from the pharmacologic and diet approaches, the use of probiotics has gradually shown promising efficacy to alleviate constipation. However, an exact understanding of the underlying mechanisms of probiotic actions on alleviating constipation is still unclear and need to be explored. In this review, we propose a model, ‘probiotics in human colon’, from a bioprocess engineering perspective. This model can be interpreted as a new concept of bionic colonic bioreactor design of a human colon in vitro, in which the transport phenomena during the fermentation of chyme by probiotics can be detected. By reviewing the anatomy structure and peristalsis mode of the human colon, we have focused on the influence by probiotics on the physical properties of colonic contents during the fermentation process. We relate physical properties such as shape, water content, density, hardness, viscosity, and elasticity to constipation symptoms directly. The influences on the physical properties of colon contents triggered by probiotics can be a potential key to understand the mechanisms for alleviating constipation.

## 1. Introduction

Constipation is a common gastrointestinal motility disorder mainly characterized by difficult defecation [1]. The primary symptoms of constipation generally present as prolonged gut transit time, reduced stool frequency, and fewer bowel movements [2]. There is a high prevalence of constipation among the population worldwide. Approximately 10–15% of people suffer from constipation according to the Rome criteria [3]. Constipation usually brings negative effects on quality of life due to both economic and humanistic burdens to the patients [4]. In terms of the causes, constipation can be subsumed under two main categories: somatopathic constipation and functional constipation. Somatopathic constipation is caused by diseases in the recto–anal area, nervous system, or endocrine system. Functional constipation can be due to a prolonged colon passage, defecation disorder, poor intake of fluids or dietary fibers, medication, or situational factors [5]. This review focuses on functional constipation, that is, conditions with no organic lesions.

Routine therapies for constipation include laxatives and diet management, but these strategies are often complained about by patients due to a lack of efficacy [6]. In recent years, accumulated evidence has shown that oral administration of probiotics is a promising approach to ameliorate constipation. Particularly, some strains belonging to *Lactobacillus* and *Bifidobacterium* provide satisfactory effects [7,8]. Up to now, the mechanisms of alleviating constipation by probiotics have received wide attention. Studies suggest that probiotics might work on regulating the gut dysmotility by impacting the gut microbiota, thus interacting with the intestinal barrier, intestinal immune system, and nervous system [9]. Furthermore, release of beneficial metabolites, such as short-chain fatty acids (SCFAs), is another crucial way for probiotics to achieve functions during the fermentation process in colon [10]. These results have provided adequate evidence on the efficacy of alleviating constipation and how probiotics perform in regulating the physiological functions of the intestinal tract, even to the whole body. However, how do these beneficial metabolites work? Why does the balance of intestinal microbiota help to alleviate constipation symptoms? What are the effects of probiotics on colonic contents? Answers to such questions remain indistinct. Hence, a more direct approach and an exact understanding of the underlying mechanisms of probiotic actions on alleviating constipation still need to be explored.

In this review, the typical probiotic strains and their curative effects on constipation compared to pharmacologic or diet approaches are summarized. A model of colon probiotics, named ‘probiotics in human colon’, from a bioprocess engineering perspective, has been proposed by us, in which the space and time of probiotic effects are analyzed. We focus on the fermentation process via probiotics based on digested chyme after the stomach and small intestine phases along with the formation of feces in the human colon. This is a dynamic physiological process involving the contact of gut microbiota and colonic contents, the transmission of semi-finished or -formed feces by colonic movement, the accumulation and storage of feces in the colon cavity, and the discharge of feces controlled by the sphincter. In order to further understand these physiological processes, we try to abstract them into an engineering model consisting of a series bionic colonic operating units with the thinking and processing method of fermentation technology. With the help of this ‘probiotics in human colon’ model, we may replicate these human physiological processes in vitro and design experiments to better explore the role and specific mechanisms of probiotic behaviors in the future. During the process of fecal formation, we consider that the physical properties of the colon contents play significant roles in the difficulty degrees of bowel movements and defecation. Hence, we pay attention to the relationship between the probiotic activities and the physical properties of colonic contents, and discuss how probiotic actions affect constipation and their treatments in this review. The physical properties of colon contents, such as shape, water content, density, hardness, viscosity, and elasticity, are thought to be more direct factors. The effects of probiotic actions on the physical properties of colon contents are considered to be the prospective mechanisms in alleviating constipation by the different probiotic species. This review may provide some new ideas on the forward steps that are necessary to be taken to benefit from the probiotic effects. These may lead to further explorations of the relationship between probiotics and human health in general, especially promoting studies using in vitro approaches on the mechanisms of probiotic effects and the treatment of diseases in the human intestinal tract.

## 2. Symptoms and Causes of Constipation

### 2.1. Definitions of Constipation

Constipation can be explained from different aspects, such as clinical criteria, diagnosis from specialists, public perceptions, and so forth. Different definitions are based on various contexts and certain emphases, thus corresponding to playing respective roles in giving diagnosis and treatments.

Clinical symptoms of constipation are usually estimated according to the Rome criteria. In Rome IV criteria issued in 2016, diagnosis of functional constipation is made as below: (1) Must include two or more of the following: straining during more than one-fourth of defecations, lumpy or hard stools in more than one-fourth of defecations, sensation of incomplete evacuation in more than one-fourth of defecations, sensation of anorectal obstruction/blockage in more than one-fourth of defecations, manual maneuvers to facilitate in more than one fourth of defecations, and fewer than three spontaneous bowel movements per week. (2) Loose stools are rarely present without the use of laxatives. (3) Insufficient criteria for irritable bowel syndrome [1]. Compared with Rome III criteria, Rome IV criteria updated the definition for functional gastrointestinal disorders and paid attention to gut–brain interactions. These disorders are classified by gastrointestinal symptoms related to any combination of motility disturbances, visceral hypersensitivity, altered mucosal and immune function, gut microbiota, or central nervous system processing [11].

From the view of specialists, infrequent bowel movements were perceived as important for diagnosing constipation based on a cross-sectional survey [12]. On the other hand, public perceptions of constipation are not always identical to clinical criteria or those by specialists. In fact, a large part of self-reported individuals did not fully match the Rome criteria, and the population suffering from constipation might be much larger than the current clinical data. It is relatively easier to provide a diagnosis of the absence of constipation for both doctors and by general population. It is, however, difficult to identify the positive diagnosis of present of constipation correctly based on Rome IV criteria. It has been revealed that unless all six symptoms of the Rome IV criteria were present, only 39–73% of individuals are correctly diagnosed with constipation [12]. Another study indicated a high rate of missed diagnosis of coexistent fecal incontinence and constipation; among the patients with self-reported coexistent symptoms meeting Rome IV criteria, only 13.6% were recognized by the referrers, while the other 86.4% were missed [13].

Definitions of constipation need to consider many related factors, and modifications of these definitions are often needed. It is important and practical if one can unify the diagnostic criteria of the symptoms of constipation with an aim of developing more precise treatment for improving the life quality and well-being of the patients.

### 2.2. Colon Movement and Constipation

The adult human colon is approximately 1.5 m in length and has an average luminal diameter of 50 mm [14]. An elongated or redundant colon are related to the pathogenesis of constipation [15,16]. In addition to absorption of water and certain nutrients, the main function of the colon is to achieve the effective propulsion of luminal contents and the eventual expulsion of residual fecal material during defecation [17].

Completion of the colonic function is a complex physiological process which involves coordinated interactions between muscular and neuronal elements. The colon receives intrinsic neural innervation from the enteric nervous system, extrinsic sympathetic innervation from the lumbar nerves, and extrinsic parasympathetic innervation from the vagus nerve in proximal colon and pelvic splanchnic nerves, which collectively govern the sensorimotor function of the colon [15,17]. The colon movement patterns could be described as segmentation and mass movement. Segmentation is stimulated by intestinal chyme which distends the haustra and leads to muscle contraction, pushing the contents to the next pouch. By mass movements, the colon contents were pushed from the transverse colon toward the rectum [14]. In 1 h before spontaneous defecation for healthy adults, both propagating sequence frequency and non-propagating activity increased, and the spatial and temporal relationships among propagating sequences demonstrated a biphasic pattern [18]. In the 15 min period preceding defecation, antegrade propagating contractions in the colon increase in frequency and amplitude. The site of origin of propagating contractions also migrates during this period, with each subsequent propagating event commencing at a more proximal location. These propagating contractions are associated with the urge to defaecate [18]. The other way around, disruption of colon movement may result in abnormal propulsion and expulsion, thus leading to constipation. It is noted that it is seldom mentioned how different portions of colon work in terms of solids and liquid transfer.

### 2.3. Physical Property of Human Feces

Apart from colon movement capacity, the physical property of the colon contents poses critical effects on fecal propulsion. The physical characteristics of human feces vary widely depending on individual health and diet. Penn et al. reviewed that the shape of human feces was from ‘hard lump’ to ‘watery diarrhea’ (‘hard lump sausage’ and ‘loose smooth snake’ are normal forms); the volume was 90–169 mL for women and 82–196 mL for men, the density was 1.06–1.09 kg/L, and the viscosity was 3.5–5.5 Pa·s [19]. In general, the solid contents of the fresh human feces ranged from 10% to 60% (*w*/*w*), corresponding to a storage modulus between 1 Pa and 10^5^ Pa approximately [20].

Solid contents contributed to the rheological quantities significantly, for example, viscosity and critical stress minimum shear stress (the minimum shear stress required to break the reticular structure) increase with increasing solid concentration in the sludge [21]. Solids in human feces consist of organic and inorganic components; the remainder and the amounts are affected by the diet supply in the population. The organic component usually contained undigested carbohydrates, fiber, proteins, and fat [22]. Studies showed that high organic contents were related to high viscosity [21]. Fresh human feces, of which the water contents ranged from 58.5% to 88.7% (*w*/*w*), had a yield stress, and the apparent viscosity decreased with the increasing shear rates. For any given shear rate, higher apparent viscosities were associated with lower water contents. Human feces are thixotropic in behavior, and the shear rheological properties of fresh human feces follows the Power Law [23,24].

In human feces, bacterial biomass is the major component of the organic fraction, which takes up 25–54% (*w*/*w*) of dry solids [22]. Fecal microbiota can reflect the community structure of colon bacteria to a certain extent. Metabolisms of the colon bacteria impact the composition of the colon contents, causing variations in the physical properties of the colon contents. Among the bacteria, probiotics has shown promising effects on constipation, which are related to normal gut motility [2]. This is because they are advised to be taken in a routine, so they are replenished in the human gut. However, the exact mechanisms of alleviating constipation by probiotics are still uncertain.

## 3. Effective Probiotic Strains on Alleviating Constipation

### 3.1. Probiotic Strains

Among various therapeutic methods on constipation, probiotics, especially some strains belong to *Lactobacillus* and *Bifidobacterium*, are gaining popularity and are even recommended by medical practitioners [8]. Up until now, dozens of probiotic species have been found to be effective against constipation based on human clinical trials and animal experiments (Table 1). Earlier studies noticed the effects of alleviating constipation by probiotic fermented milk. Milk fermented with *Streptococcus thermophilus*, *Lactobacillus acidophilus*, and *Bifidobacterium infantis* significantly reduced constipation symptoms including defecation duration, urgency, straining, stool consistency, and feelings of incomplete evacuation, while better effects were obtained when fermented milk was mixed with dietary fiber [25]. The common commercial yogurt fermented by *Lactobacillus bulgaricus* and *S. thermophilus*, even after pasteurizing, was found to be effective in alleviating constipation symptoms due to SCFAs produced by the probiotics [26]. A modified fermented yogurt containing *Lactococcus lactis*, *Lactobacillus plantarum,* and *Lactobacillus casei* could significantly increase defecation frequency and improve intestinal health, as well as change the intestinal bacterial community by increasing probiotic bacterial numbers [27]. Kefir-fermented milk was also found effective to alleviate constipation for the patients with mental and physical disabilities, but this benefit varies depending on individuals, since some showed no effect [28]. It has been reported that *Lactobacillus kefiranofaciens* is the main dominant species among Kefirs of different origins [29]. Fermented milk containing *L. casei* Zhang and *Bifidobacterium animalis* ssp. *lactis* V9 also showed good effects of alleviating constipation. It was speculated that this probiotic fermented milk may contribute to regulating gastrointestinal microbiota, fighting inflammation, and regulating metabolic pathways [30].

Apart from the fermented milks, other probiotic foods are also available. For example, fermented black garlic by *L. plantarum* X7022, played roles in promoting small intestinal peristalsis via measuring the length from the pylorus to the forefront of fed ink on constipated mice, which significantly decreased defecation time, increased defecation number, defecation weight, and small intestine transit ratio [31]. Combination of konjac glucomannan and *Lactobacillus paracasei* X11 [32], a combination of konjac glucomannan and *B. animalis* F1-7 [33], can both promote defecation in constipated mice by increasing the SCFA metabolism and 5-hydroxytryptamine (5-HT) hormone release.

Compared to the complex system of fermented foods, it is relatively easier to detect the functional substances from a single bacterial strain. A randomized controlled trial showed *Bifidobacterium longum* BB536 improved defecation and some upper abdominal symptoms in elderly patients with chronic constipation [34]. Another randomized controlled trial indicated alleviation effects of constipation from *L. plantarum* P9 intervention was accompanied by desirable changes in the human feces via fine tuning of certain functional intestinal microbiota, bacteriophages, and microbial metabolites [35]. *L. plantarum* Lp3a was reported to alleviate functional constipation symptoms by enhancing intestinal motility, which was putatively associated with methane metabolism and bile acid synthesis; but no significant difference was detected in microbiome composition, which suggests that the observed effect of *L. plantarum* Lp3a on constipation was not directly mediated by the microbial diversity [36]. An experiment on mice showed that *Lacticaseibacillus paracasei* NCU-04 effectively improved the depression-like behaviors associated with constipation, possibly through 5-HT-mediated microbiota–gut–brain axis [37]. *L. lactis* subsp. *Lactis* HFY14 can inhibit constipation by regulating mRNA expression of the VIP-cAMP-PKA-AQP3 signaling pathway, which increased serum levels of neurotransmitters including motilin, gastrin, endothelin, acetylcholinesterase, substance P, and vasoactive intestinal peptide, and decreased serum levels of somatostatin in constipated mice [38]. In recent years, SCFAs have been concerned as important effective metabolites produced by probiotic strains. Intaking *B. animalis* subsp. *lactis* MN-Gup contributed to regulating the gut microbiota, where the relative abundances of acetate-producing *Bifidobacterium*, *Ruminoccaceae_UCG-002*, and *Ruminoccaceae_UCG-005* were significantly increased [39]. *Bifidobacterium bifidum* G9-1 improved constipation parameters, such as defecating frequency, fecal water content, and fecal hardness in constipated rats, which was considered to be related with the concentrations of butyric acid and neurotransmitters [40]. Contrary to previous studies, five *Lactobacillus rhamnosus* strains, CCFM1068, FFJND15-L2, FHeNJZ7-1, FTJDJ11-1, and FZJHZ11-7, were found to improve constipation symptoms to various degrees, but their abilities were not associated with the levels of SCFAs in the colon, while the authors suggested that the effects were associated with gastrointestinal regulatory peptides, neurotransmitters, neurotrophic factors, and gut microbiota [41]. Apart from concentrating on SCFAs in most studies, Zhang et al. indicated the potential from long-chain fatty acids, especially stearic acid (C18:0) produced by *B. longum* S3 [42].

Not only lactic acid bacteria, but also some *Bacillus* strains have also been reported to be effective. A double-blind placebo-controlled study in human adults confirmed *Bacillus coagulans* Unique IS2 can reduce the symptoms of constipation, such as stool consistency, difficulty of defecation, defecation, and abdominal pain [43]. *Bacillus subtilis* CBD2 and *B. subtilis* KMKW4 positively affected the intestinal function by suppressing the growth of pathogenic microbiota and the activity of undesired enzymes as well as ameliorating constipation, since these two strains inhibited the activity of β-glucosidase and tryptophanase, identified as harmful enzymes of the intestinal microbiota [44].

Some researchers have focused on multi-strains to alleviate constipation. The 4-week supplementation of *S. thermophilus* MG510 and *L. plantarum* LRCC5193 significantly improved stool consistency in patients with chronic constipation, and the beneficial effect of *L. plantarum* on stool consistency remained after the probiotic supplementation was discontinued, which indicated colonization and reproduction of *L. plantarum* [45]. Five mixed strains, including *L. plantarum*, *L. acidophilus*, *B. bifidum*, *Bifidobacterium lactis*, and *S. thermophilus*, showed a constipation-alleviating effect; these effects were mediated by improving the intestinal environment and SCFA production [46]. Probiotics used with prebiotics showed better effects in alleviating constipation than using probiotics alone, but the addition of prebiotics did not affect the total SCFA content significantly [46]. Mixed usage of *B. animalis* subsp. *lactis* HN019, *Lacticaseibacillus rhamnosus* HN001, and fructooligosaccharide suggested the potential roles of enteric neurohormone and 5-HT in the probiotic-derived optimizations in gut microbial genera, related to bowel movement in humans [47]. According to the above results, the effective actions of multiple species are similar to that of single species. In multi-strain studies, whether a single bacterium plays a dominant role or the mixed strains work together is unknown. Whether the effect of multi-strains is better than that of single strain is worth further investigation.

Furthermore, improving bowel movement is not always equal to counteracting constipation. A randomized controlled trial showed that *Lactobacillus reuteri* DSM17938 is effective for improving bowel movements and bodyweights in pediatric patients with anorexia nervosa and constipation, but the difference was not statistically significant in the alleviation of constipation [48]. The mechanisms in alleviating constipation by probiotics still need more precise and persuasive explanations.

**Table 1:** Main effective probiotic strains on constipation.

Strains	Subjects	Doses	Main Findings	References
*Bifidobacterium longum* S3	Mice	0.2 mL of bacterial suspension (5 × 10^9^ CFU mL^−1^) for 4 weeks.	*B. longum S3* exerted a constipation-alleviating effect primarily by improving the gut microbiota, repairing intestinal barrier damage and reducing inflammation, as well as inhibiting oxidative stress levels and decreasing expression of water channel proteins.	[42]
*Lacticaseibacillus paracasei* NCU-04	Mice	1 × 10^8^ CFU/d or 1 × 10^9^ CFU/d for 14 days.	*L. paracasei* NCU-04 significantly enhanced gut immobility, reduced colon inflammation, and increased levels of colonic motilin, 5-HT and c-kit. Notably, *L. paracasei* NCU-04 effectively upregulated the expression of 5-HT and its receptor in the brains of constipated mice.	[37]
*Bifidobacterium longum* BB536	Humans (elderly)	2 g/package (5 × 10^10^ CFU or more), 1 sachet daily for 4 weeks.	The randomized controlled trial showed *B. longum* BB536 improved defecation and some upper abdominal symptoms in elderly patients with chronic constipation. Some of the improved symptoms were maintained even 4 weeks after stopping the probiotics.	[34]
*Bifidobacterium animalis* subsp. *lactis* HN019 + *Lacticaseibacillus rhamnosus* HN001 + fructooligosaccharide	Humans (adults)	HN019: 7.7 × 10^9^ CFU/sachet; HN001: 1.9 × 10^9^ CFU/sachet; Fructooligosaccharide: 0.48 g/sachet. 2 sachet every day for 4 weeks.	The results suggested the potential roles of enteric neurohormone and 5-HT in the probiotic-derived optimizations in gut microbial genera, related to bowel movement in human.	[47]
*Lactiplantibacillus plantarum* P9	Humans (adults)	2 g per sachet, 1 × 10^11^ CFU/sachet/day for 28 days.	The constipation alleviation effect of *L. plantarum* P9 was fine tuning of certain functional intestinal microbiota, bacteriophages, and microbial metabolites.	[35]
*Lactobacillus plantarum* Lp3a	Humans	1.0 × 10^10^ CFU per bag, 2 bags per day for 7 days.	*L. plantarum* Lp3a can alleviate functional constipation symptoms by enhancing intestinal motility, which is putatively associated with methane metabolism and bile acid synthesis. 16s rRNA sequencing analysis revealed no significant difference in microbiome composition, which suggests that the observed effect of *L. plantarum* Lp3a on constipation is not directly mediated by microbial diversity.	[36]
	Mice	10–30 times of the human daily dose for 15 days.		
Fermented black garlic by *Lactobacillus plantarum* X7022	Mice	20 mL/kg bodyweight, once a day for 14 days.	The defecation time was significantly decreased, and the defecation number and the defecation weight were increased. Fermented black garlic by *L. plantarum* X7022 played roles in promoting small intestinal peristalsis on constipated mice.	[31]
*Lactococcus lactis* subsp. *Lactis* HFY14	Mice	1 × 10^9^ CFU/kg for 4 weeks.	*Lactococcus lactis* subsp. *Lactis* HFY14 inhibits constipation by regulating mRNA expression of the VIP-cAMP-PKA-AQP3 signaling pathway.	[38]
Mixture of probiotics and prebiotics Probiotics: *Lactobacillus plantarum*, *Lactobacillus acidophilus*, *Bifidobacterium bifidum*, *Bifidobacterium lactis*, *Streptococcus thermophilus*; Prebiotics: Lactitol, Kamut steamed powder, Microbiome X.	Rats	LACTO 5X: 31 mg/kg, Synbiotics: 120 mg/kg, once daily for 21 days.	The use of multi-strain probiotics alone showed a constipation-alleviating effect, but probiotics used with prebiotics showed better effects in alleviating constipation than using probiotics alone.	[46]
Combination laxative: konjac glucomannan + *Lactobacillus paracasei* X11	Mice	*L. paracasei* X11: 1.0 × 10^8^ CFU/mL at 0.6 mL/d, konjac glucomannan: 10 mg/mL, for 17 days.	Konjac glucomannan and *L. paracasei X11* effectively promoted the metabolism of SCFA and the secretion of 5-HT in mice. Up-regulated mRNA and protein levels of 5-HT receptor 4 and serotonin transporter via the 5-HT pathway effectively alleviated constipation.	[32]
Combination laxative: konjac glucomannan and *Bifidobacterium animalis* F1-7	Mice	5 mL of konjac glucomannan (0.015 g/15 mL) + 5 mL of *B. animalis* F1-7 (2 × 10^8^ CFU/mL), 10 mg/kg per day for 21 days.	Konjac glucomannan + *B. animalis F1-7* mixture effectively improved intestinal motility and alleviated constipation through humoral transport-related pathways. The mixture effectively promoted defecation in mice, increased the fecal water content, shortened the defecation time and improved the gastrointestinal transit rate. Secretion of 5-HT and SCFA were also promoted.	[33]
*Bifidobacterium animalis* subsp. *lactis* MN-Gup	Mice	2 × 10^9^ cfu/kg, once per day for 14 days.	*B. animalis* subsp. *lactis* MN-Gup significantly decreased the first black stool defecation time, and significantly increased black fecal wet weight, black fecal number and the gastrointestinal transit rate, thereby alleviating constipation.	[39]
	Humans	10^10^ CFU per day for 4 weeks.	Concentration of acetate significantly increased. MN-Gup can alleviate constipation related to increased acetate-producing *Bifidobacterium*, *Ruminoccaceae_UCG-002* and *Ruminoccaceae_UCG-005*.	
*Bifidobacterium bifidum* G9-1	Rats	1.0 × 10^10^ CFU, three times a day for 4 days.	*B. bifidum* G9-1 improved dysbiosis and prevented a decrease in butyric acid concentration in the gut, increased serum serotonin, and suppressed an increase in dopamine and a decrease in acetylcholine in serum, while increased the expression level of tryptophan hydroxylase 1, a 5-HT-synthetizing enzyme.	[40]
*Lactobacillus reuteri* DSM17938	Humans (children with anorexia nervosa)	10^8^ CFU, twice daily for 3 months.	*L. reuteri* DSM17938 is more effective than placebo for improving bowel movements and weight normalization in anorexia nervosa pediatric patients with constipation. The number of patients alleviated from constipation was higher in the *L. reuteri* than in the placebo group (87% vs. 63%), but the difference was not statistically significant.	[48]
*Bacillus coagulans* Unique IS_2_	Humans (adults)	2 × 10^9^ CFU, once daily for 4 weeks.	In the probiotic treated group, significant increase was observed in number of bowel movements, symptoms of incomplete evacuation, painful defecation and abdominal pain associated with constipation were alleviated. 98% subjects in the probiotic group achieved normal stool consistency as compared to placebo (74%).	[43]
*Lactobacillus rhamnosus* CCFM1068; *Lactobacillus rhamnosus* FFJND15-L2; *Lactobacillus rhamnosus* FHeNJZ7-1; *Lactobacillus rhamnosus* FTJDJ11-1; *Lactobacillus rhamnosus* FZJHZ11-7.	Mice	0.2 mL of bacterial suspension (5 × 10^9^ CFU/mL in 3% sucrose solution) daily for 18 days.	Five strains of *L. rhamnosus* can alleviate constipation-related symptoms via different pathways independent of SCFAs regulation. The effects were associated with gastrointestinal regulatory peptides, neurotransmitters, neurotrophic factors, and gut microbiota.	[41]
Fermented milk containing *Lactobacillus casei* Zhang and *Bifidobacterium animalis* ssp. *lactis* V9	Humans (adults)	200 g/d of fermented milk for 4 weeks.	After intervention, the anti-inflammatory cytokine IL-10 increased and the proinflammatory cytokine C-reactive protein and lipopolysaccharides decreased. *B. animalis* was correlated with an increase in defecation frequency. Acylcarnitine, located on the significantly altered carnitine shuttle pathway, had a significantly positive correlation with defecation frequency.	[30]
Chocolate containing *Streptococcus thermophilus* MG510 *and Lactobacillus plantarum* LRCC5193	Humans (adults)	*S. thermophilus*: 3.0 × 10^8^ CFU/g, *L. plantarum*: 1.0 × 10^8^ CFU/g, once daily for 4 weeks.	The relative abundance of *L. plantarum* was significantly greater in the probiotic group than in the placebo group, the relative abundance of *S. thermophilus*, *Bifidobacterium* spp., *Bacteroidetes* and *Firmicutes* did not change significantly in either group during the study period. The levels of serum cytokines (IL-10/IL-12 ratio and TNF-α) did not differ significantly between the two groups.	[45]
Fermented yogurt containing *Lactococcus lactis*, *Lactobacillus plantarum* and *Lactobacillus casei*	Mice	10^6^ CFU/mL, 4 mL per day for 5 days.	A new formulation of yogurt could significantly improve defecation time and intestinal health. Yogurt intake could also increase probiotic numbers and change the intestinal bacterial community composition.	[27]
Pasteurized yogurt fermented with two strains of *Lactobacillus bulgaricus* and two strains of *Streptococcus thermophilus*	Humans (adults)	No living bacteria (<5 CFU/mL)	Pasteurized yogurt, with inactive lactic acid bacteria, was found to be effective in improving defecation frequency and constipation symptoms. The numbers of fecal bifidobacteria and lactobacilli, and the SCFA concentrations increased.	[26]
*Bacillus subtilis* CBD2; *Bacillus subtilis* KMKW4	Mice	1 × 10^7^ CFU per day for 7 days.	*B. subtilis* CBD2 and KMKW4 strains positively affect the intestinal function by suppressing the growth of pathogenic microflora and the activity of harmful enzymes as well as ameliorating constipation.	[44]

### 3.2. Probiotics Versus Pharmacologic Approaches

Compared to probiotics, the use of laxatives is an ordinary pharmacologic approach to treat constipation. Based on the mode of action, laxatives are commonly classified as bulk-forming laxatives, osmotic laxatives, stimulant laxatives, secretagogues, and others [5,49].

Psyllium husk is a representative and effective bulk-forming laxative, which is not only a traditional medicine in China and India, but also high-quality source of dietary fiber in the food industry [50]. The gelatinous mass of psyllium husk helps increase fecal volume and produce soft stool [51]. Limited digestible oligosaccharides in psyllium husk can provide great effects on intestinal microbial composition and alterations in the levels of acetate and propionate [52]. To this extent, psyllium husk works within a similar field to probiotics and can be considered as prebiotics. It has been reported that synthetic fresh fecal sludge containing psyllium husk had a higher dewaterability than fresh fecal sludge, which is likely due to the high water-binding affinity of the psyllium husk [19].

Common examples of osmotic laxatives are lactulose and polyethylene glycol. These laxatives are named because they can increase the osmotic pressure in the colon lumen and retain water in the intestine, and thus soften the stool and trigger the reflex action of the bowel peristalsis [5]. Interestingly, we can see that modes of the effects of lactulose and psyllium are similar. Recent studies reveal that lactulose was also associated with gut microbiota, since the bacteria can break down lactulose into SCFAs [53]. Deng et al. compared the efficacies of lactulose and a Chinese herb solid drink made from psyllium husk, hemp seed, sweet almond, black sesame, and resistant dextrin; they indicated that the Chinese herb had a better effect in elevating the levels of propionic acid, butyric acid, isobutyric acid, valeric acid, isovaleric acid, and hexanoic acid, while lactulose had a better effect on elevating the levels of acetic acid in the feces [54]. Comparing the use of lactulose with probiotics, the effects of *Lactococcus lactis* subsp. *lactis* HFY14 alleviating constipation symptoms were found to be similar to those of lactulose [38]. Polyethylene glycol, another common osmotic laxative, promotes defecation by increasing colonic water content, which can interact with water molecules by forming hydrogen bonds in a ratio of 100 water molecules per 1 polyethylene glycol molecule [55].

Stimulant laxatives or secretagogues may be used if there is no response to osmotic laxatives. Stimulant laxatives, such as bisacodyl, stimulate the intestinal mucosa and nerve plexus to secrete water and electrolytes [56]. Secretagogues, such as lubiprostone, act directly on intestinal epithelial cells, increasing fluid secretion into the intestinal cavity [57]. Both stimulant laxatives and secretagogues can increase intestinal water contents, resulting in peristaltic contraction and reductions in the transit time in the colon. Generally, stimulant laxatives and secretagogues are used for short-term emergent treatment due to side effects including diarrhea and abdominal pain; the longer-term effects of these drugs and their safety are unknown.

Although laxatives were used both for the treatment and prevention of constipation, laxatives are often perceived to be ineffective, and healthcare professionals do not always suggest laxatives for treatment [58]. Satisfaction levels with laxatives are reported to be low (28–50%) because laxatives may not always be effective [59].

**Figure 1:**
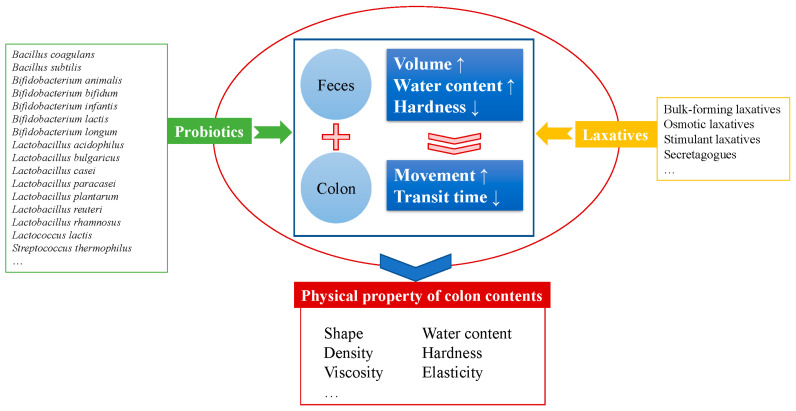
An intersection of alleviating constipation using probiotics and laxatives.

As shown in Figure 1, probiotic and pharmacologic approaches are presented for alleviating constipation. The goal of the two approaches is considered to be similar, i.e., the alteration of the physical properties of colon contents such as shape, water content, density, hardness, viscosity, and elasticity. More precisely, the actions of both probiotics and laxatives can be attributed to possibly alter the conditions of feces and the colon. As described above, some probiotic strains which are capable of producing SCFAs contributed to enhancing fecal volume, increasing the water content of feces, as well as decreasing fecal hardness. Similarly, osmotic laxatives and stimulant laxatives are related to higher water content and lower hardness of feces, while bulk-forming laxatives are effective in improving fecal volume. In terms of the colon, SCFAs and 5-HT probiotic producers are able to stimulate colon movement and result in the reduced intestinal transit time of the feces. In the same way, stimulant laxatives and secretagogues can also achieve this. The specific measures acting on the feces or colon are consolidated and complement one other. They all intend to achieve the common goal of alleviating constipation. In essence, the key point is the alternation of the physical properties of the colon contents and feces.

### 3.3. Probiotics Versus Food

Based on similar performances to probiotic and pharmacologic approaches, some natural foods are also reported to be effective in alleviating constipation, especially fruits that are rich in dietary fiber. Dragon fruit oligosaccharide, a non-digestible, fermentable, and soluble short-chain carbohydrate, increased colonic smooth muscle contractions without morphological change and acted as a bulk-forming and stimulant laxative to increase fecal output and intestinal motility [60]. Chey et al. compared the effectiveness of green kiwifruit, psyllium, and prunes among the patients with chronic constipation in America; the results indicated that prunes and psyllium led to significant and sustained increases in stool frequency over the 4-week treatment period [61]. The authors also confirmed green kiwifruit to be a safe, effective, and well-tolerated treatment for a subset of patients with chronic constipation. Katsirma et al. summarized the fruits that have been shown to modify the microbiota or have an impact on gut motility, among which prunes, raisins and apple fiber isolate increased fecal weight in humans, kiwifruit increased small bowel and fecal water content, while apple fiber isolate, kiwifruit, fig paste, and orange extract reduced the gut transit time [62].

Remarkably, since the probiotics, pharmacologic, and diet approaches served similar functions and contributed to alleviating constipation with similar substantial basis, it can be considered that there may be a common mechanism behind the effects. With this idea, we are trying to reveal the intrinsic connections between probiotic behaviors and their effects in the human colon from an engineering perspective in order to extend the research methods of conventional animal or clinical experiments and provide research directions based on in vitro fermentation innovatively.

## 4. A Model of ‘Probiotics in Human Colon’ from an Engineering Perspective

### 4.1. Illustration of the ‘Probiotics in Human Colon’ Model

**Figure 2:**
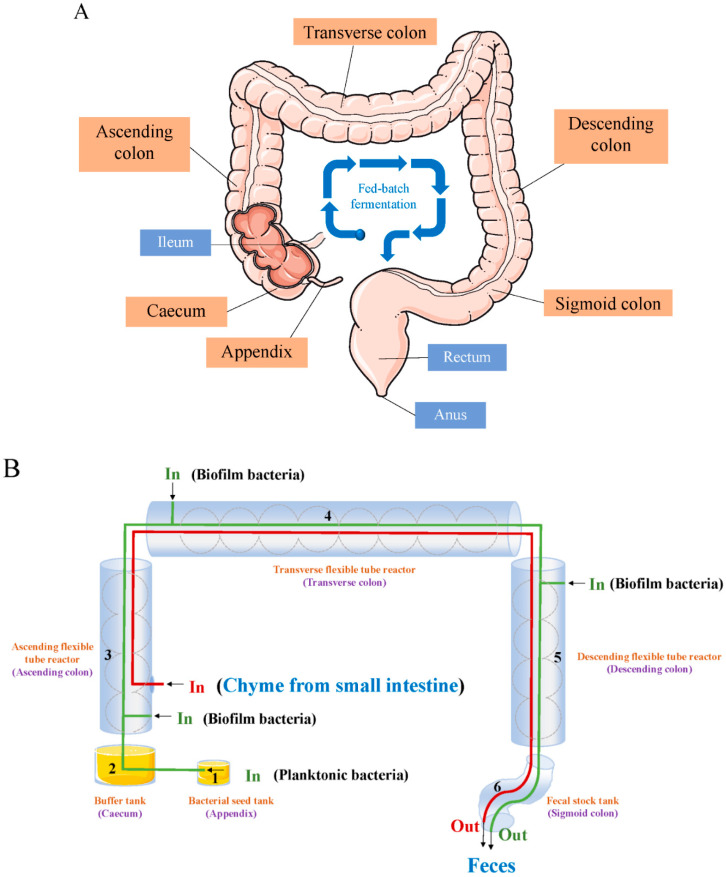

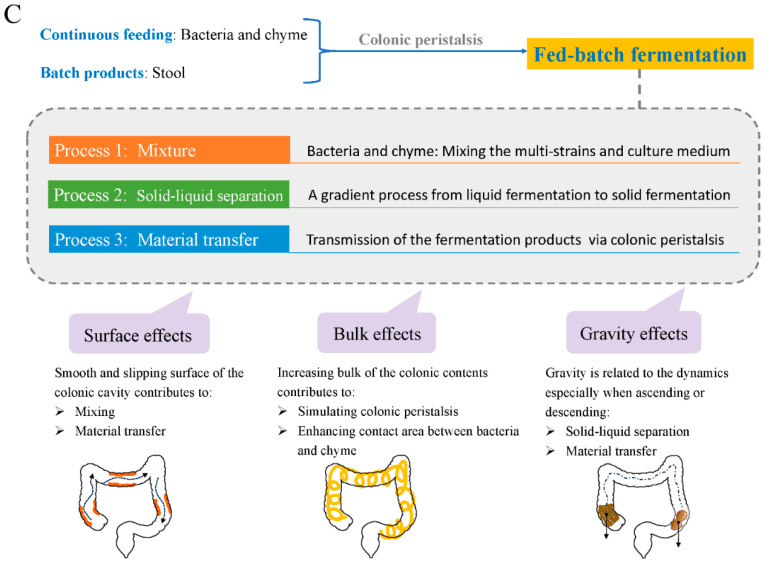
Human colonic fermentation analysis from on engineering perspective. (**A**) Schematic of human colon structure; (**B**) design of the colonic fermentation process in a bionic bioreactor; (**C**) typical engineering processes and important factors in the colonic fed-batch fermentation.

**Figure 3:**
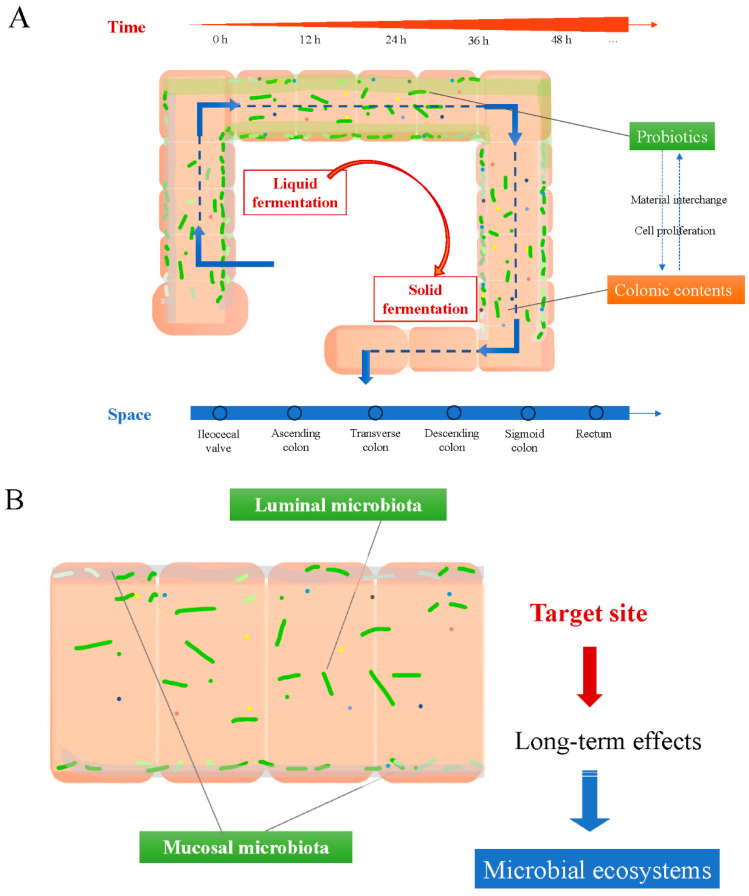

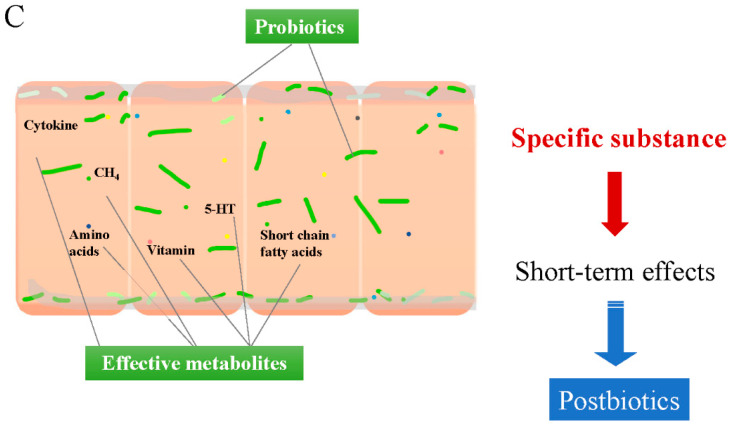
A model of ‘probiotics in human colon’ from a bioprocess engineering perspective. (**A**) Schematic of probiotic effects in the space and time dimensions during upstream–downstream process; (**B**) target site for the probiotics to come into play in the long term; (**C**) specific substance produced by the probiotics to be effective in the short term.

As presented in Figure 2 and Figure 3, based on the literature survey and some intuitive reasoning, we have proposed a model of the colonic probiotics from an engineering perspective. The model ‘probiotics in human colon’ is expected to provide a visualized in vitro approach for detailing the mechanisms of probiotic actions in the human colon and initiating attentions to the prospective mechanisms in alleviating constipation by probiotics.

Designed based on real human colon conditions, as shown in Figure 2A, the chyme, after small intestinal digestion, is transmitted from the ileum into the ascending colon. Potentially acting as an important source of bacterial strains, the caecum harbors adequate microbiota similar to the colon in both humans and animals [63,64]. The bacteria in the lumen are mixed with chyme from ileum, then passed though along the ascending colon, transverse colon, and descending colon. During this process, bacterial biofilm colonized within the colonic mucosa also participates in the fermentation of chyme and formation of feces [65,66]. Feces are gradually accumulated and stored temporarily in the sigmoid colon, then excreted periodically via the rectum and anus. Thus, this process can be regarded as fed-batch fermentation. Particularly, the role of the appendix is still unclear and controversial. The appendix has always been considered as a useless vestige along the history of human evolution; however, some studies indicated that the appendix may largely be favorable to extended life span in mammals [67]. It is reported the appendix is an immunologically rich and active organ with broad composition similar to that of the colonic microbiome, but with distinct taxonomic proportions [68].

In our view, as illustrated in Figure 2B, the fermentation process in the human colon is summarized and transformed into biochemical reactions in a bionic bioreactor from an engineering perspective. We regard the appendix as a bacterial seed tank of gut microbiota, including probiotics, where concentrated strains of various species are stored. The caecum is an easily ignored, but very important unit; as first proposed in our previous article [69], caecum could be a buffer tank, which connects the upper stream operation in ileum and the downstream operation in the ascending colon. In this buffer tank, the planktonic bacteria are amplification cultured, similar to the process in a secondary seed fermenter. There are two approaches for the colonic bacteria into this set of bioreactors: one is through the seed tank and buffer tank as for planktonic bacteria, and the other is the release of colonized biofilm bacteria on the inner wall of ascending, transverse, and descending colon. Meanwhile, the entrance of simulated chyme from small intestine, acting as bacterial medium, is designed in the intersection between the ileum and the ascending colon. The ascending, transverse, and descending colons would be made into flexible tube reactors, where solids and liquids are mixed and transmitted by haustration movement and mass peristalsis. After the fed-batch fermentation, the feces are produced and stocked in the sigmoid colon.

As summarized in Figure 2C, we propose three typical bioprocess and engineering processes in the fed-batch fermentation. (1) Mixture: The multi-species bacteria (both planktonic and biofilm bacteria) and chyme in the colon are mixed—that is, the bacterial seeds are inoculated into the culture medium intermittently. For a laboratory bionic bioreactor, the planktonic bacteria are cultured at the seed tank and buffer tank, while the biofilm can be immobilized cultured within a thin film made from solid medium coated on the inner surface of the flexible tube reactors. (2) Solid–liquid separation: Since the colon absorbs water in the digestive products, the moisture content would be gradually decreasing during fermentation, which can be considered as an operating unit of solid–liquid separation. This process of solids and liquid transfer corresponds to the gradual formation of feces from food chyme and bacteria within the colon lumen. After finishing the digestion stage in the small intestine, the food chyme is then delivered to the colon. At this point, the digesta is mixed in intestinal fluid, of which the volume is several times the initial volume of food. The digesta is continuously squeezed and passed through the colon during a stepwise process of dehydration—that is, the separation of solid and liquid. The water content of the mixture decreases to approximately 50% when the feces are finally formed. (3) Material transfer: With colonic peristalsis, the fermentation products via bacteria are transported from the ascending, transverse, descending colon to the sigmoid colon. During these processes, we point out three important factors including surface effects, bulk effects, and gravity effects. (a) Surface effects: The properties of the colonic inner wall, such as smoothness and slippage, affect the mixing and transport of the colonic contents. (b) Bulk effects: Increasing the bulk of the colonic contents will simulate colonic peristalsis and enhance the contact area between bacteria and chyme. Considering laboratory settings to create more practical guidelines for bioreactor design, the volume of the colonic contents can be used as a measurable parameter to indicate the bulk effects. (c) Gravity effects: Due to gravity, solid–liquid separation in the ascending colon may be easier, while fecal transit in the descending colon is more favorable. This is an excellent example for morphology’s influence on the dynamics, and a trend that should be paid attention to in the design of fermentation bioreactors. Under a same extrusion force, the difference value of material transfer distance between the ascending colon and transverse colon, descending colon, and transverse colon, respectively, can be used to measure the gravitational effects.

Different from in vivo conditions, this assumption about the colonic bioreactor will help to break through the physiological and ethical limitations and obtain samples of colonic contents at any time point and site when conducting scientific experiments. This will be of great scientific and practical value for studying the mechanisms of action of probiotics in the human colon. Of course, there are also unavoidable limitations to this in vitro model. The initial establishment of intestinal bacterial ecosystems in vitro is extremely complex and time-consuming, and many species cannot be cultured in vitro yet due to the limitations of existing technologies.

As shown in Figure 3A, then we further focus on the effects from probiotics among the complex colonic bacterial community. The ‘probiotics in human colon’ model, with biomimetic significance, paid attention to the space and time dimensions of probiotic effects, which concerned the continuous and dynamic actions during the upstream–downstream process from an engineering perspective. In human colons, fermentation of the chyme by microbiota, including probiotics, may generally last for 0.5–3 days according to defecation frequency. With the increase in fermentation time, the fermentation process was accompanied by the transport of spatial location. The digests entering from the ileocecal valve were successively transported to the ascending colon, transverse colon, descending colon, sigmoid colon, and rectum, and are finally discharged through the anus. This upstream and downstream relationship is very important in the dynamic fermentation process because the influence of the preceding process on the subsequent process is accumulated over time and space.

A crucial but still unclear issue is whether the main effects are performed on the inner surfaces of human colon by mucosal microbiota or inside the colon cavity by luminal microbiota. It is important to know which plays a dominant role between the viable bacteria cells and their metabolites in order to explore the exact mechanisms probiotics use for their effects on intestinal disease. From our perspective, the target site for the probiotics to come into play may make for the probiotic actions in the long term (Figure 3B), while specific effective substances produced by the probiotics are related to their actions in the short term (Figure 3C). Mucosal microbiota, colonized on the surface of the colon inner wall, might mainly dependent on living bacteria by maintenance of ecological balance. Luminal microbiota, mixed inside the colon cavity, might mainly depend on effective metabolites such as SCFAs, amino acids, vitamins, 5-HT, cytokine, methane, and hydrogen. Those two action modes, long term and short term, are further discussed below.

### 4.2. Long-Term Effects from Colonized Probiotics on the Colonic Mucosa

When the probiotics colonized on the inner surface of colon, their behaviors can be associated with the bacterial community. Colonized probiotic community functions as a holistic organization and interacts with the host, which may affect the structure of indigenous microbiota and provide sustainable and dynamic benefits to human health. The colonization of probiotics is related to long-term effects for human body. The role of viable bacteria is long term, although the active ingredients are unknown. Individual differences may exist, and the functions are usually affected by the physiological conditions of the host.

A big challenge for colonization of the supplementary probiotics is the presence of an endogenous microbiota that occupies the ecological niche [70]. In view of this issue, fecal microbiota transplantation (FMT) is used as an untargeted microbiome modulation technique, which transplants the fecal microbiota of healthy people into the gastrointestinal tract of patients in order to reconstruct their intestinal microbiota [71]. FMT has been proved to be therapeutic efficient in alleviating slow-transit constipation [72]. Donor microbiota was reported to persist 3 months after FMT treatment, with extensive coexistence of the recipient strains [73]. In spite of this, there is still an obvious insufficiency that needs to be solved, considering the implementation of FMT in clinical practice. FMT results indicated that young-age mice, of which the gut microbiota had not been established stably, favored the donor microbiota engraftment and removal of endogenous microbiota [74]. For mice experiments, germ-free mice can be employed, or the depletion of recipient gut microbiota could also be depleted by antibiotics or polyethylene glycol to achieve better engraftment [75]. But systematic human clinical data on the efficacy and long-term and short-term adverse reactions of FMT are still inadequate, especially determining and monitoring the improvement or deterioration response to FMT of the recipients [72].

A significant benefit of probiotic colonization in the human colon is to produce antagonistic effect against pathogens and maintain balance of intestinal microbiota through competitive metabolism activities such as nutrient scramble and bacteriocin secretion [76,77]. Wu et al. hold a view that gut bacteria in the complex ecosystem may work together as coherent functional groups called guilds, which may be derived from exploiting the same resources in a similar way or possessing certain interactive molecular mechanisms [78]. Co-occurring communities are polarized in the competition–cooperation landscape with different metabolism and fitness, and competitive communities can better resist species invasion, while cooperative communities are resilient to nutrient change [79]. Gut microbiome can be modulated by interventions of probiotics. Research results proved that the gut microbiota of mice was significantly altered at the genus level after supplying with probiotic compounds [80]. Metabolism and biotransformation actions of the harmonious probiotic community in the intestinal track are associated with health-promoting properties, including maintenance of the gut barrier function and modulation of the host immune system [81]. Research findings also suggest that mechanisms for constipation effects by probiotic bacteria are multifactorial, involving anti-inflammatory and immunomodulatory functions [2]. More than a few commensal bacteria, especially some next-generation probiotic strains, possess the ability to protect the epithelial barrier and interact with the host immunology tells, playing an essential role in maintaining gut homeostasis against microbiota dysbiosis [82].

A limiting factor in investigating mechanisms of probiotic colonization is the source of colon bacterial samples. Due to the difficulty in obtaining clinical intestinal bacteria samples, most clinical data are based on fecal bacteria samples. Several studies assessed the ability of probiotic bacteria to reach the gut and colonize it by evaluating their fecal recovery, but this colonization ability is highly strain-dependent, which explained why many data conflict on the fecal recovery of probiotic bacteria [83]. Monteagudo-Mera et al. reviewed adhesion mechanisms by probiotic bacteria to the host and also indicated that the intake of probiotics could lead only to a temporary colonization; hence, they suggest that more in vitro and in vivo studies are needed to elucidate the mechanisms and clinical efficacy of probiotics [84].

### 4.3. Short-Term Effects from Supplemented Probiotics Inside the Colonic Cavity

When the probiotics are merely mixed in the colon contents inside the colonic cavity, their functions might be attributed to their beneficial metabolites. These effects are short-term, which lasts within the period ranging from the arrival of probiotics in the colon to subsequent defecation. In order to further reveal the mechanisms in alleviating constipation by probiotics, investigations on the substantial basis of probiotic effects are indispensable. Different from results of viable probiotic supplementary, it has been reported that inactive lactic acid bacteria, taking pasteurized yogurt as an example, was also effective in improving constipation [26]. The effective roles of inactivated bacteria against constipation are related to their bioactive compounds that worked in the colon. The health beneficial compounds synthetic by probiotics mainly involves SCFAs, amino acids, and peptides, vitamins, antioxidant activities, anti-inflammatory, and immune-modulating compounds and exopolysaccharides [85].

SCFAs have attracted plentiful attentions among the bioactive compounds produced by probiotics in colon due to their varieties of excellent probiotic functions including curing constipation. SCFAs, mainly including acetate, propionate, and butyrate, are derived from the hydrolysis of non-digestible carbohydrates, such as polysaccharides, oligosaccharides, and disaccharides [86]. Mice experiments showed that alleviation of constipation can be achieved by acetic acid and butyric acid based on increased small intestinal transit rate and water content of feces, but propionic acid did not show a significant effect [87]. 

During the process of carbohydrate hydrolysis, several kinds of gases including carbon dioxide, methane, and hydrogen can be released together with SCFAs [86]. However, methane has been considered to be related with constipation because it was able to slow down small intestinal transit [88]. It is reported that patients affected by constipation produce higher methane, whereas patients with chronic non-specific diarrhea have a higher excretion of hydrogen in breath samples [89]. A study showed *L. reuteri* DSM 17938 presented beneficial effects on chronic constipation via a significant decrease in methane production [90]. But there are a few studies questioned these associations because they found that constipation and bloating severity did not always correlate with methane levels on glucose breath testing [91]. The reason for this disagreement is that the mechanism of association between methane and constipation is unclear, and the effects from the gases on properties of colon contents still need further explorations. Combining the information above to the ‘probiotics in human colon’ model, the volumes and types of gas produced by the microorganisms can be determined during an in vitro experiment, when the fermentation process is carried out in the bionic colonic bioreactor.

Elevated proteolytic metabolites have also been reported to be strongly associated with colonic transit time and constipation. Among Parkinson patients with gastrointestinal dysfunction, the gut microbiota was characterized by reduced carbohydrate fermentation and butyrate synthesis capacity and increased proteolytic fermentation and production of deleterious amino acid metabolites, including p-cresol and phenylacetylglutamine, which inhibited colonocyte oxidative respiration and proliferation [92].

It is reported that oral intake of Vitamin B_12_ helps to decrease the risk of digestive disorder and constipation [93]. Probiotic bacteria are able to produce critical nutrients and growth factors, the most important of which are vitamin B_12_, which is not synthesized by plants, and vitamin K, which helps the growth and establishment of the useful bacteria in the gut [85].

Probiotics also help to alleviate constipation via regulation of inflammation. Fermented milk containing *L. casei* Zhang and *B. animalis* ssp. *lactis* V9 significantly benefited constipation, the anti-inflammatory cytokine IL-10 increased and the proinflammatory cytokine C-reactive protein, and lipopolysaccharides decreased in the serum samples after intervention [30]. Mice experiments indicated that probiotic compounds enhanced the abnormalities of substance P, motilin, and gastrin, and reduced the abnormalities of vasoactive intestinal peptide, somatostatin, and endothelin, thereby regulating gastrointestinal regulatory hormones and alleviating constipation [80].

Overall, the metabolites, together with probiotics, may improve host health uniformly. In recent years, the definitions of the term ‘postbiotics’ were proposed based on a comprehensive look at probiotics and their metabolites. Postbiotics comprise metabolites and/or cell-wall components released by probiotics [94]. In 2019, the International Scientific Association for Probiotics and Prebiotics (ISAPP) convened a panel and defined a postbiotic as a ‘preparation of inanimate microorganisms and/or their components that confers a health benefit on the host’ [95]. Compared to probiotics, postbiotics possess several advantages such as follows: availability in their pure form; ease in production and storage; availability of production process for industrial-scale-up; specific mechanism of action; better accessibility of Microbes Associated Molecular Pattern (MAMP) during recognition and interaction with Pattern Recognition Receptors (PRR); and more likely to trigger only the targeted responses by specific ligand-receptor interactions [96]. Development of postbiotic products has shown valuable potential for commercial application in the functional foods market [97,98].

The role of bacterial metabolites is short-term, and it is relatively easy to detect the exact functional component and effective dosage. On the one hand, specific hormones produced by the probiotics stimulated colon movement, thus decrease intestinal transit time. On the other hand, beneficial metabolites alter physical properties of colon contents, such as volume, water content, hardness, and viscosity of feces, thus helping to alleviate constipation directly. We consider that the physical properties of colon contents play a direct and crucial role, which link up probiotic actions and constipation symptoms. Impacts from probiotic behaviors on the physical properties of colon contents can be a prospective mechanism in alleviating constipation. Apart from constipation, in essence, we assume that the final destination of probiotics might lie in developing colon-targeted supplements with a defined composition and dosage, which is a research idea based on a drug development for human health.

## 5. New Insights into the Mechanisms of Alleviating Constipation by Probiotics Related to the Physical Properties of Colon Contents

**Figure 4:**
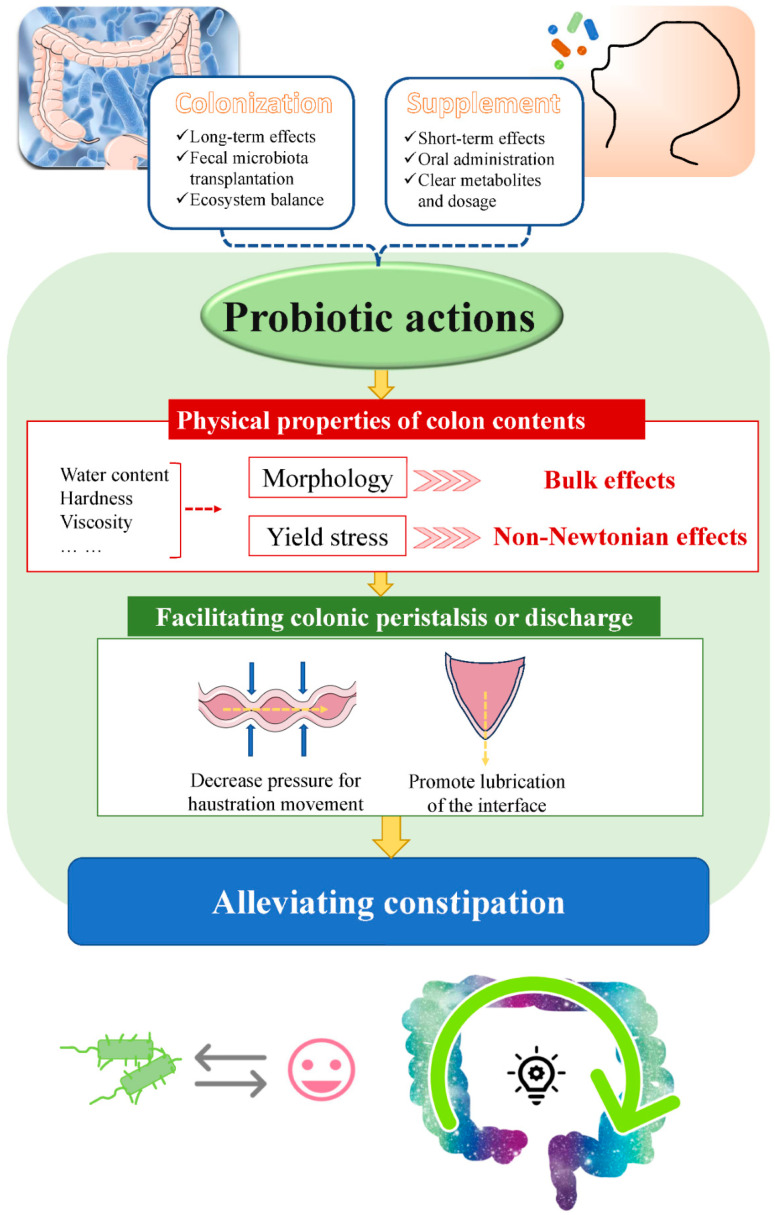
New insights into the mechanisms of alleviating constipation by probiotics related to the physical properties of colon contents.

In this review, as demonstrated in Figure 4, we emphasize that the direct causal association between constipation symptoms and probiotic actions should be well studied. We have a view that changes in the physical properties of colon contents are directly responsible for constipation. The effects of probiotic actions on the physical properties of colon contents before forming feces are considered to be the prospective mechanisms in alleviating constipation by the different probiotic species. Different probiotic species have different influences based on various substances and functions summarized above; a common intersection of the effective treatments by probiotics might point to physical properties of colon contents such as shape, water content, density, hardness, viscosity, and elasticity, particularly the rheology properties. The bacterial metabolites, as well as their production process in the colon, might affect the physical properties of colon contents directly. Variations in the physical properties of colon contents pose impacts on the consistency of defecation. The critical factors on the physical properties of colon contents associated with constipation symptoms are still unrevealed and are worth investigating.

Shape and water content: According to ‘Bristol Stool Form Scale’, stools can be classified into seven items related to their shapes and water contents, and a change in stool form score correlates with a change in whole-gut transit time [99]. The colon is the final area where electrolytes and water are absorbed before excretion; a complex interaction between secretory and absorptive processes maintains the electrolyte homeostasis [100]. Colonic epithelial cells are responsible for maintaining this delicate balance between luminal secretion and the absorption of fluids and ions [101]. The equilibrium states of fluids and ions decide water content of colon contents, thus contributing to the shape and water content of feces. It has been reported that SCFAs stimulate colonic blood flow and fluid and electrolyte uptake [86]. Several novel bacterial compounds and peptide hormones related to heat-stable enterotoxin and guanylin were also found to act upon electrolyte and water balance [102].

Density and hardness: The density and hardness are associated with the solid compositions of colon contents. An earlier study revealed a curvilinear correlation between percent insoluble stool solids and stool hardness; hardness increased only slightly as percent insoluble solids increased between 7% and 20%, but hardness increased dramatically when percent insoluble solids exceeded 25% [103]. For formula-fed infants, calcium and fatty acid soaps were the dominant factors significantly related to stool solids and hardness [104]. Particularly, unabsorbed palmitic acid tended to react with calcium to form insoluble soaps, while well-absorbed *sn*-2-palmitate formulas contributed to reduced stool soaps [105,106]. For children and young adults, an intake of dietary fiber helped to decrease stool hardness and constipation symptoms [107,108]. Abnormally high levels of stool hardness are not common in constipated subjects [103]. The density and hardness of feces can be affected by dietary factors, but the stool samples could not present a complete picture of colonic situations; more dynamic and precise investigations on colon contents are still expected. For laboratory settings using the bionic colonic bioreactor, the density of feces can be calculated by measuring weight and volume, and the hardness of feces can be detected using a texture analyzer. It is meaningful to establish standardized measurement techniques from bioprocess engineering, which is expected to provide appropriate methods for in vitro colonic fermentation and to produce comparable, reproducible experimental data.

Viscosity and elasticity: During anaerobic fermentation, organic compounds, including proteins, carbohydrates, and lipids, usually undergo hydrolysis, acidification, and methanation. The hydrolysis of polysaccharides by intestinal microbiota can increase the viscosity and thixotropy of the mucus system and the interfacial strength [109]. Analogously, in fermentation of waste activated sludge, the SCFA content increased and the viscosity of the waste sludge decreased [110]. The solubility and viscosity of different fiber sources were correlated to in vitro fermentation kinetics, total gas production, and SCFA profile [111]. The influence of viscosity on the growth of human gut microbiota were evaluated, and the results show that changes in intestinal viscosity could lead to selective modification of microbiota composition, mainly due to their aerobic or anaerobic properties of the microorganism [112]. Furthermore, the yield stress of feces increased exponentially with their solid content (from 20 Pa to 8000 Pa), and difficulties in defecation may result either from unduly high yield stress or pathologies of reflex recto–anal dilatation or a combination of the two [113].

These findings provide evidence for the role of the physical properties of the colon contents on constipation by probiotics. Because probiotic actions affect the physical properties of the colon contents, subsequently, changes in colon contents in shape, water content, density, hardness, viscosity, elasticity, and so forth are helpful in decreasing the pressure for colonic peristalsis, thus alleviating constipation. We consider that this is a more direct and refined step further into the mechanisms of constipation, which deserves to be understood clearly. In future studies, physical properties may be meaningful indicators for the quantitation of diagnosis for treatment of constipation. Exploitation of a standard range for physical properties of colon contents can be beneficial to human health and life quality. At present, there is limited evidence on these properties of colon contents after probiotic interventions, and this deserves investigation in future studies. This may also be related to the systemic, comprehensive, and complex physiological state of human body, which is connected to various factors and relies on mutual coordination and restriction.

## 6. Conclusions

This review provided insights into a novel model of ‘probiotics in human colon’, which is a new concept for designing a bionic colonic bioreactor. Via this model, the mechanisms of alleviating constipation by probiotics were discussed innovatively. ‘Probiotics in human colon’ is established based on a bioprocess engineering perspective, and is proposed for the first time with a focus on the space and time of probiotic effects. We consider the effects of probiotic actions on the physical properties of colon contents, particularly density and hardness, as the prospective mechanisms in alleviating constipation by different probiotic species. This is a further essential analysis of the underlying mechanisms of the role of probiotic actions. In future studies, several indistinct directions are expected to be addressed, including the role of the physical properties of colon contents for human intestinal health, including, but not limited to, giving an engineering quantitation of the probiotic efficacy on the ground of specific parameters, achieving accurate control of probiotic functions, and exploring more thorough mechanisms of beneficial behaviors of probiotic bacteria. This information is important for the future development of colon-targeted probiotic products in the field of human health.

## Data Availability

No new data were created or analyzed in this study. Data sharing is not applicable to this article.
